# Cloning and Functional Characterization of Octβ2-Receptor and Tyr1-Receptor in the Chagas Disease Vector, *Rhodnius prolixus*

**DOI:** 10.3389/fphys.2017.00744

**Published:** 2017-09-26

**Authors:** Sam Hana, Angela B. Lange

**Affiliations:** Department of Biology, University of Toronto Mississauga, Mississauga, ON, Canada

**Keywords:** octopamine, tyramine, G-protein-coupled receptor, antagonists, insect

## Abstract

Octopamine and tyramine, both biogenic amines, are bioactive chemicals important in diverse physiological processes in invertebrates. In insects, octopamine and tyramine operate analogously to epinephrine and norepinephrine in the vertebrates. Octopamine and tyramine bind to G-protein coupled receptors (GPCRs) leading to changes in second messenger levels and thereby modifying the function in target tissues and insect behavior. In this paper, we report the cDNA sequences of two GPCRs, RhoprOctβ2-R, and RhoprTyr1-R, have been cloned and functionally characterized from *Rhodnius prolixus*. Octopamine and tyramine each activate RhoprOctβ2-R and RhoprTyr1-R in a dose-dependent manner. Octopamine is one order of magnitude more potent than tyramine in activating RhoprOctβ2-R. Tyramine is two orders of magnitude more potent than octopamine in activating RhoprTyr1-R. Phentolamine and gramine significantly antagonize RhoprOctβ2-R, whereas yohimbine and phenoxybenzamine are effective blockers of RhoprTyr1-R. The transcripts of both receptors are enriched in the central nervous system (CNS) and are expressed throughout the adult female reproductive system. It has been shown in other insects that Octβ2-R is essential for processes such as ovulation and fertilization. We previously reported that octopamine and tyramine modulate oviducts and bursa contractions in *R. prolixus*. Our data confirm the importance of octopamine and tyramine signaling in the reproductive system of *R. prolixus*.

## Introduction

Biogenic amines are a class of organic neuroactive chemicals, derived from amino acids, characterized by having low molecular weights and an amine moiety and are an integral component of neuronal communication and signaling in animals. Biogenic amines are utilized by neurons to send quick, private and short-term signals to specific targets leading to transient physiological changes. Octopamine, a biogenic amine, is not only known to be a neurotransmitter, but also to act as a neuromodulator and a neurohormone in insects (Orchard, 1982; Roeder, 1999; Farooqui, 2012). Octopamine's precursor, tyramine, is also known to be an independent neuroactive chemical signaling through tyramine specific receptors (Kononenko et al., 2009; Lange, 2009). In insects, the octopaminergic system functions in a similar manner to the adrenergic system in vertebrates (Roeder, 1999, 2005). Octopamine and tyramine play a variety of physiological roles in insects, thereby modulating feeding (Cohen et al., 2002; Ishida and Ozaki, 2011), learning and memory (Hammer and Menzel, 1998; Schroll et al., 2006), aggression (Zhou et al., 2008; Szczuka et al., 2013), locomotion (Saraswati et al., 2004) and metabolism (Orchard et al., 1993; Scheiner et al., 2002).

**Figure 1:**
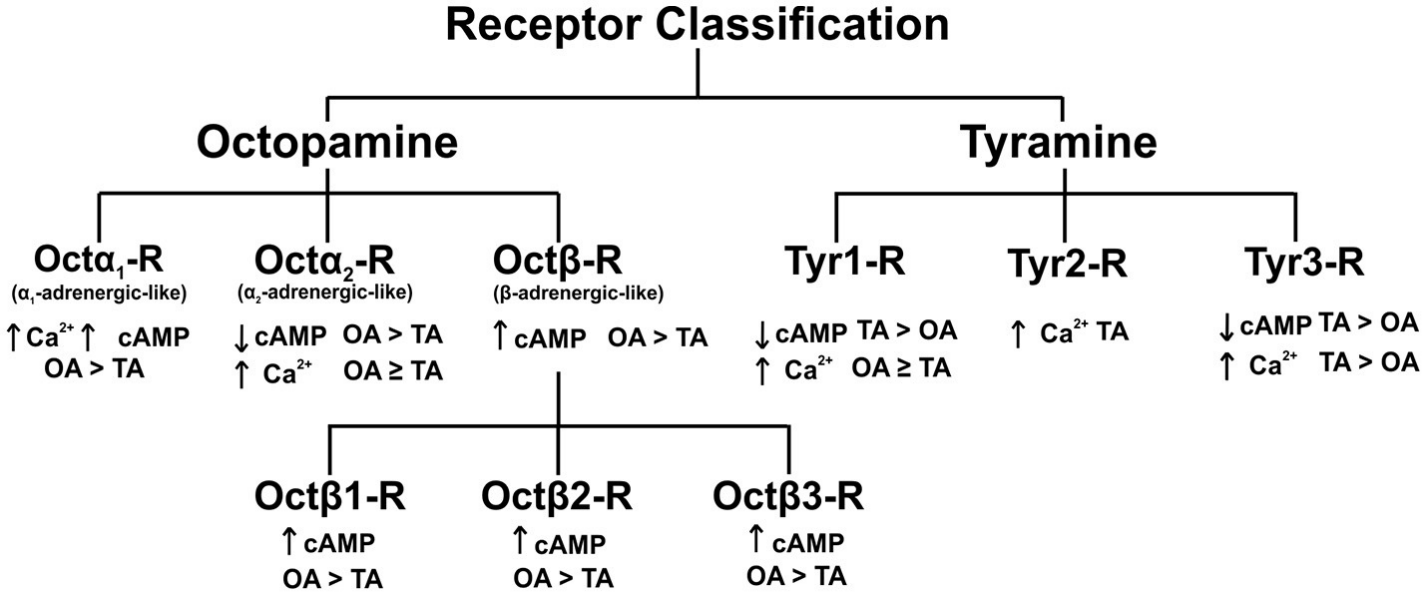
Classification of octopamine and tyramine receptors in insects. The recently discovered octopamine α_2_-adrenergic-like receptors and tyramine 3 receptor in *D. melanogaster*. OA, octopamine; TA, tyramine; Ca^2+^, calcium; cAMP, cyclic adenosine monophosphate. Figure based on Wu et al. (2014).

Octopamine and tyramine utilize G-protein coupled receptors (GPCRs) on post-synaptic or target tissue membranes to induce physiological effects. The first receptor cloned and characterized was a *D. melanogaster* tyramine receptor shown to negatively couple to adenylate cyclase (Arakawa et al., 1990). Fast forward 27 years later, many octopamine and tyramine receptors have been cloned and characterized from different insect orders, most notably in Diptera, Lepidoptera and Hymenoptera (Ohta and Ozoe, 2014; Wu et al., 2015; Reim et al., 2017). The activation of a specific receptor leads to a unique change(s) in cAMP and/or Ca^2+^ as depicted in Figure 1. Insect octopamine and tyramine receptors are now classified into Octα_1_-R, Octα_2_-R, Octβ-Rs (Octβ1-R, Octβ2-R, Octβ3-R), Tyr1-R, Tyr2-R, and Tyr3-R (Evans and Maqueira, 2005; Farooqui, 2012; Wu et al., 2014; Figure 1). The signaling pathways, and classification of these receptors has been defined using a variety of bioassays and second messenger analysis along with studies using specific agonists and antagonists (Evans and Maqueira, 2005; Farooqui, 2012; Wu et al., 2014).

Octopamine and tyramine signaling pathways have been shown to be essential in modulating the reproductive system of various insects. For example, lack of tyramine and octopamine in *Drosophila melanogaster* (tyrosine decarboxylase 2 mutated flies) resulted in reproductive sterility due to egg retention (Cole et al., 2005). Insects that specifically lack octopamine (tyramine β-hydroxylase mutants) accumulated eggs in their ovaries due to abolished ovulation (Monastirioti et al., 1996; Monastirioti, 2003). A tyramine 1 (Tyr1) receptor in *Locusta migratoria*, the octopamine receptor in the mushroom bodies (OAMB) and octopamine beta 2 (Octβ2) receptor in *D. melanogaster*, have also been linked to reproductive physiology in both insects (Lee et al., 2003, 2009; Donini and Lange, 2004; Molaei et al., 2005; Lim et al., 2014; Li et al., 2015). A recent study demonstrated Octβ2-R knockdown hindered ovulation in *Nilaparvata lugens* (Wu et al., 2017). Octopamine and tyramine may exert some of their effects by influencing the contractions of the reproductive musculature. Thus, octopamine reduces the amplitude, frequency and basal tonus of lateral oviduct contractions in *D. melanogaster* (Middleton et al., 2006; Rodriguez-Valentin et al., 2006), *L. migratoria* (Lange and Orchard, 1986), and *Stomoxys calcitrans* (Cook and Wagner, 1992).

*Rhodnius prolixus* is a blood-feeding hemipteran and the vector of Chagas disease in Central and South America. It has previously been shown that octopamine reduces the amplitude of spontaneous and RhoprFIRFamide (AKDNFIRFamide)-induced oviduct contractions in a dose-dependent manner (Hana and Lange, 2017). Tyramine did not reduce spontaneous oviduct contractions, but did decrease RhoprFIRFamide-induced oviduct contractions suggesting that the action of tyramine is via modulation of induced contractions (Hana and Lange, 2017). At the bursa, a large muscular sac responsible for oviposition in *R. prolixus*, octopamine and tyramine reduce the frequency of contractions at ≤10^−7^ M and abolish contractions at ≥10^−6^ M (Hana and Lange, 2017). Thus, octopamine/tyramine signaling may be important in the reproductive system of this medically-important insect.

The present study aimed to confirm this by identifying and functionally characterizing two receptors, RhoprOctβ2-R and RhoprTyr1-R, in *R. prolixus*. RhoprOctβ2-R and RhoprTyr1-R are shown to be dose-dependently activated by their corresponding ligand. Both receptors are widely distributed throughout the adult female reproductive system suggesting vital roles in modulating reproductive processes. This study, not only supports octopamine's role in reproduction, but also suggests that tyramine can be involved in the fine-tuning of reproductive processes in *R. prolixus*. The newly discovered *R. prolixus* octopamine and tyramine receptors could potentially be used to develop lead compounds to be used as vector control.

## Materials and methods

### Animals

Adult *R. prolixus* were maintained with a 12 h light/dark cycle reared at ~50% humidity and 28°C and fed defibrinated rabbit's blood (Hemostat Laboratories, Dixon, CA, USA; supplied by Cedarlane Laboratories Inc., Burlington, ON, Canada) once in every instar.

### Chemicals

All biogenic amines (D, L-octopamine hydrochloride, tyramine hydrochloride, serotonin hydrochloride and dopamine hydrochloride) were made as 10^−2^ M stocks dissolved in double distilled water. All biogenic amine antagonists (phentolamine hydrochloride, gramine, metoclopramide hydrochloride, mianserin hydrochloride, cyproheptadine hydrochloride, phenoxybenzamine hydrochloride, yohimbine hydrochloride, and chlorpromazine hydrochloride) were prepared in molecular grade ethanol or dimethyl sulfoxide to 10^−2^ M stocks. The final percentage of solvent in the experimental treatments was ≤0.1%. All chemicals were obtained from Sigma Aldrich (Oakville, Canada).

### Isolation and cloning of cDNA sequences encoding *R. prolixus* Octβ2 and Tyr1 receptors

The scaffold, transcripts and proteins of *R. prolixus* were uploaded into Geneious 8.1 (Auckland, New Zealand) from vectorbase.org. Using *D. melanogaster* Octβ2-R-PA (Q4LBB9) and *D. melanogaster* Tyr1-R (Q9VEG1) as templates, a tblastn search against *R. prolixus* transcripts and proteins as performed. The partial cDNA sequences of the closest results to the query sequence obtained for Octβ2-R (RPRC011545) and Tyr1-R (RPRC008712) were amplified by specific primers for each receptor (Table S1). OneTaq® DNA Polymerase (NEB, Whitby, ON, Canada) was used for all PCRs. The cycling profiles for the PCRs using Bio-Rad's s100 thermocycler (Bio-Rad Laboratories, Mississauga, ON, Canada) were: initial denaturation at 94°C for 5 min, followed by 29 cycles at 94°C for 30 sec, 55–63°C annealing for 1 min, 68°C for 1 min and a final extension at 68°C for 5 min. Products from the reactions were gel extracted using EZ-10 Spin Column DNA Gel Extraction Kit (Bio Basic, Markham, ON, Canada) and cloned using pGEM-T Easy Vector (Promega, Madison, WI, USA). White colonies containing the inserts, tested using PCR, were inoculated and left overnight to grow. The inserts were extracted using EZ-10 Spin Column Plasmid DNA MiniPreps Kit (Bio Basic, Markham, ON, Canada) and sent for Sanger sequencing at the Centre of Applied Genomics at the Hospital for Sick Children (Toronto, ON, Canada) or Eurofins Genomics (Toronto, ON, Canada).

Gene specific forward primers (Table S2) were used along with pDNR-LIB−25 Revs plasmid primer to amplify the cDNA regions at the 3′ end of the receptors (3′ Modified RACE). The product of the first reaction was nested with another gene specific primer (Table S2). This process was repeated until one characteristic band for each receptor was observed. The bands were gel purified, cloned and sequenced. Many gene specific forward primers (see Table S3) were designed for the amplification of the 5′ cDNA ends of both receptors (5′ Modified RACE). Essentially, a series of nested PCRs were utilized to distinguish the correct bands. The products of the first reaction were purified and used as a template for the subsequent reaction. Selected fragments were gel extracted, cloned and sequenced. The cDNA sequences for RhoprOctβ2-R (MF377526) and RhoprTyr1-R (MF377527) have been deposited in GenBank.

### Sequence analysis

The seven transmembrane domains for both receptors were predicted by TMHMM Server v. 2.0 (http://www.cbs.dtu.dk/services/TMHMM/). BLAST was used to predict exon-intron boundaries for both receptors, this was confirmed by a fruit fly splice site prediction tool (http://www.fruitfly.org/seq_tools/splice.html). The intracellular and N-glycosylation sites were predicted using NeyGlyc 1.0 Server (http://www.cbs.dtu.dk/services/NetNGlyc/) and the intracellular phosphorylation sites were predicted using NetPhos 3.1 Server (http://www.cbs.dtu.dk/services/NetPhos/). Palmitoylation of cysteine residues were predicted by GPS.Lipid. An Integrated Resource for Lipid Modifications predictor (http://lipid.biocuckoo.org/webserver.php).

### Mammalian expression vectors and transfection of the receptors

The open reading frames (ORF) of both RhoprOctβ2-R and RhoprTyr1-R were amplified using Q5® High-Fidelity DNA Polymerase (New England Biolabs, Massachusetts, United States) and the Kozak translation initiation sequence (GCCACC) was inserted at the 5′ end of each receptor (Table S4; Kozak, 1987). The products were cloned into a pGEM-T Easy vector (Promega, Madison, WI, USA) and sequenced. The receptors were reamplified with Bgl II restriction site introduced at the 5′ end and Bam HI site introduced at the 3′ end of each receptor (Table S4). RhoprOctβ2-R was subcloned into pIRES2-ZsGreen1 (Clontech, Mountain View, CA, USA) and RhoprTyr1-R was subcloned into pIRES2 DsRed-Express2 (Clontech, Mountain View, CA, USA).

A HEK293/CNG cell line that stably expresses a modified cyclic nucleotide-gated channel (CNG) (previously available from BD Biosciences, Mississauga, ON, Canada) were raised in Dulbecco's Modified Eagle Medium Nutrient Mixture F12-Ham (DMEM/F-12) (Thermo Fisher Scientific, Waltham, MA, USA) supplemented with 10% heat-inactivated fetal bovine serum, 1% penicillin and streptomycin, and 100 μg/mL G418. The cells were incubated at 37°C in 5% CO_2_. The cells were grown in T75 flasks to 90–95% confluency and were transiently co-transfected with either expression vector containing the receptor and aequorin at a 2:1 ratio (transfection reagent to expression vectors) using X-tremeGENE® HP DNA Transfection Reagent (Roche Applied Science, Penzberg, Germany). The cells were incubated for 72 h and used for the functional cell assay.

### Functional cell assay

HEK293/CNG cells were harvested with PBS-EDTA solution and placed in working bovine serum albumen (BSA) medium (DMEM/F-12 with 1% BSA and 1% penicillin and streptomycin). The cells were incubated in coelenterazine h (Promega, Madison, WI, USA) to a final concentration of 5 μM with stirring in the dark for at least 3 h. Before running the bioluminescence assay, the cells were diluted by 5-fold using the BSA medium. For the dose-response curves, stock solutions of D, L-octopamine hydrochloride and tyramine hydrochloride were diluted in BSA medium and tested in triplicate in flat bottom CELLSTAR 96 well-plates (Greiner Bio-One, Kremsmunster, Austria). Using an automated injector, 50 μL of cells were loaded into each well and luminescence was measured over three intervals for 15 s using a Wallac Victor2 plate reader (Perkin Elmer, San Diego, CA, USA). The agonists, serotonin and dopamine, were tested in a similar manner as above. For the antagonists assay, octopamine or tyramine were loaded into the wells along with each antagonist, all dissolved in BSA medium. The plate was then vortexed for 2 min before running the assay.

### Spatial expression of RhoprOctβ2-R and RhoprTyr1-R in the adult female reproductive system

The expression of both receptors was examined in 1 week old, unfed female, 4 weeks post-feeding as fifth instars. The central nervous system (CNS), ovary (OVA), lateral oviducts (LOV), common oviduct and spermatheca (COS), bursa (BUR), cement (CEM) were dissected and placed in nuclease-free phosphate-buffered saline (PBS) (Sigma Aldrich, Oakville, ON, Canada). Total RNA extraction was followed using EZ-10 Spin Column Total RNA Minipreps Super kit (Bio Basic, Markham, ON, Canada) and cDNA was synthesized using High-Capacity cDNA Reverse Transcription Kit (Applied Biosystems, Mississauga, ON, Canada). The total cDNA produced was diluted by 10-fold and used for the quantitative PCR reactions. The *RhoprOct*β*2-R, RhoprTyr1-R* and the reference genes (α-tubulin, β-actin, ribosomal protein 49) were amplified by Mx35005 Quantitative PCR System (Stratagene, Mississauga, ON, Canada) using the primers provided (Table S5) and SsoFAST EvaGreen Supermix with low Rox (Bio-Rad, Mississauga, ON, Canada). The reaction conditions for both receptors started with an initial denaturation at 95°C for 30 sec followed by 40 cycles of denaturation at 95°C for 5 s, annealing and extension at 60°C for 33 s. Two technical replicates were performed per tissue along with a non-template control for each biological replicate. Analysis of relative expression levels was determined using the ΔCt method. Normalized expression was obtained by first averaging the C_T_ values of the reference genes β-actin, α-tubulin and rp49; and second comparing these values to the C_T_ values of the gene of interest in all tissues.

### Statistical analysis

GraphPad Prism version 5.03 (www.graphpad.com) was used to create and statistically analyze all graphs in this paper.

## Results

### Structure of RhoprOctβ2 and RhoprTyr1 receptors

**Figure 2:**
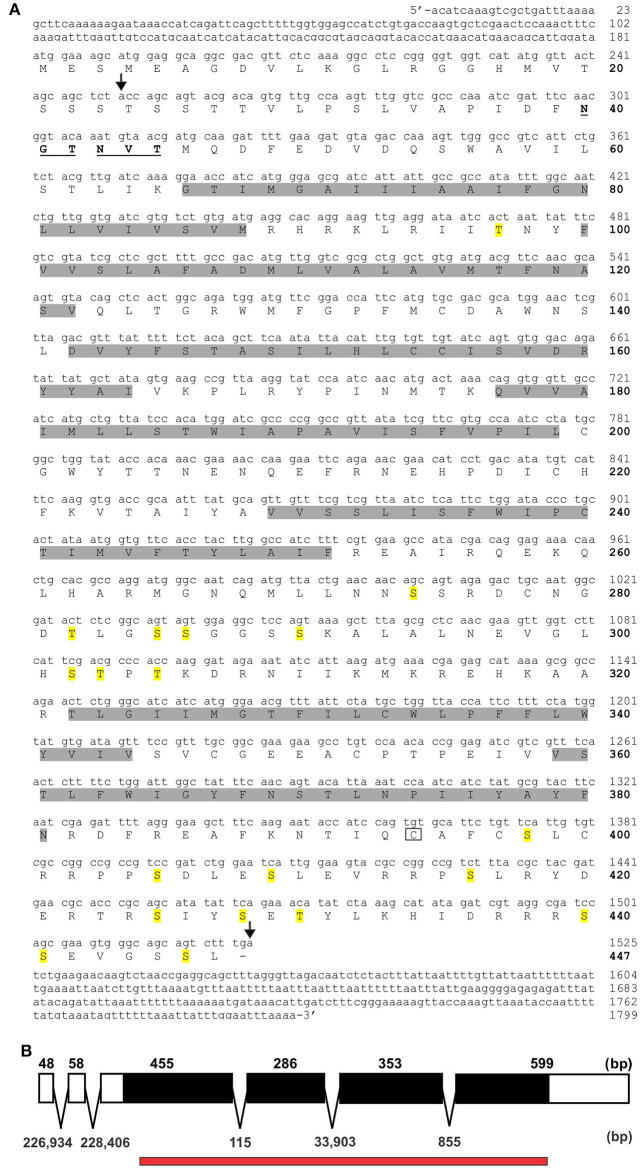
RhoprOctβ2-R cDNA sequence and the corresponding predicted amino acid sequence. **(A)** Amino acid numbers are bolded and indicated on the right of the sequences below the nucleotide numbers. The predicted transmembrane domains are highlighted in gray. The predicted N-glycosylation sites are underlined and bolded, while the potential phosphorylation sites are highlighted in yellow. The boxed cysteine residue is a site of potential palmitoylation. The region within the arrows indicate the predicted cDNA sequences from VectorBase. **(B)** RhoprOctβ2-R's open reading frame is indicated in solid black formed by four exons. The numbers above the exon map indicate exon length while the numbers below the exon map indicate intron lengths. The red bar below the exon map indicates the predicted regions obtained from VectorBase.

**Figure 3:**
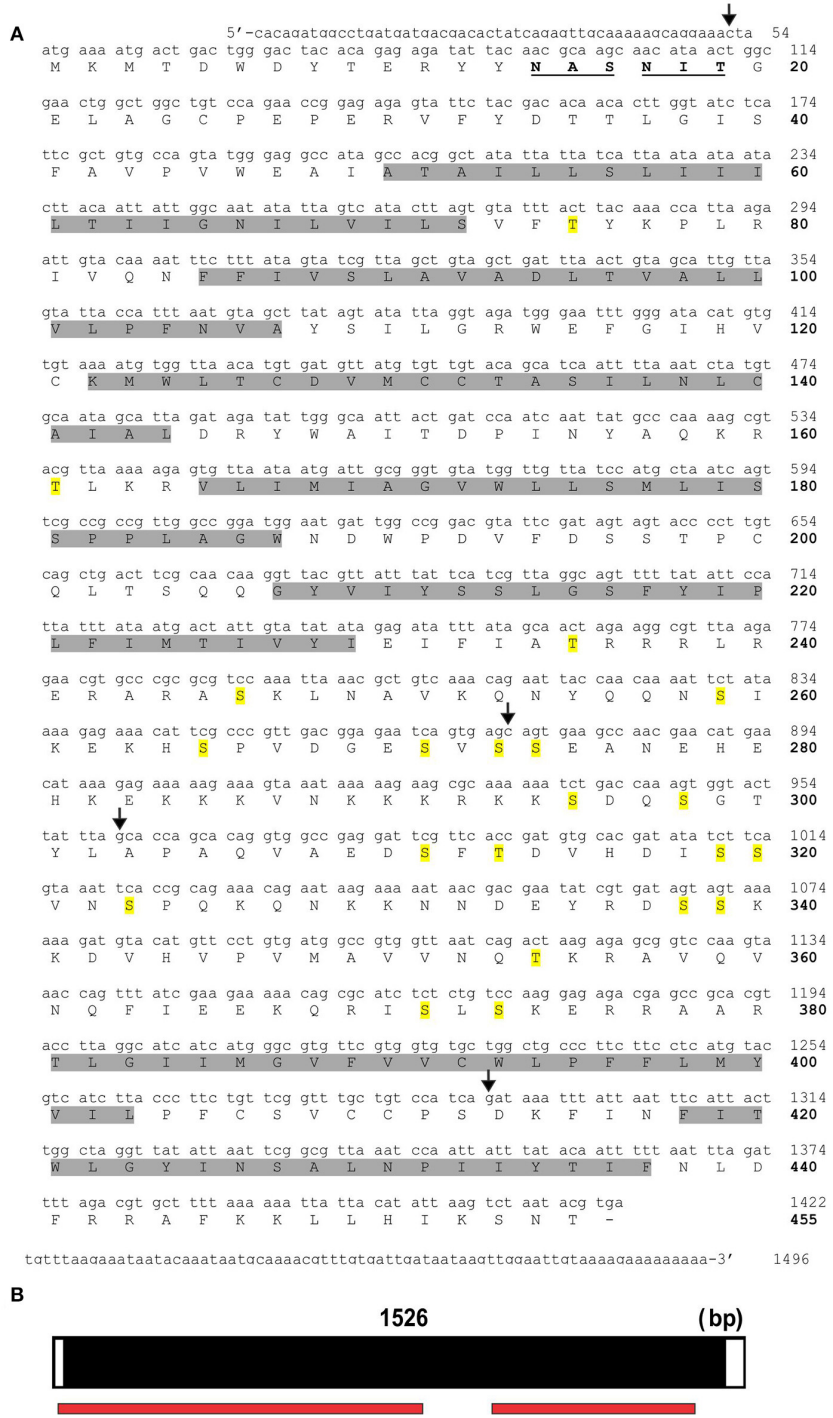
RhoprTyr1-R cDNA sequence and the corresponding predicted amino acid sequence. **(A)** The nucleotide numbers are indicated on the right above the bolded amino acid numbers. The predicted hydrophobic transmembrane domains are highlighted in gray. The predicted N-glycosylation residues are bolded and underlined. Residues highlighted in yellow indicate potential phosphorylation sites. The region within the first and second arrow + third and fourth arrow indicate predicted cDNA sequences from VectorBase. **(B)** The open reading frame of RhoprTyr1-R gene spans a single exon as indicated in black. The red bar below the exon map indicates the predicted regions obtained from VectorBase.

Various potential biogenic amine receptors were analyzed *in silico* using *D. melanogaster* receptors as templates. Two targets were selected for 5′ and 3′ amplification by PCR. The full sequences of RhoprOctβ2-R and RhoprTyr1-R were obtained by a modified method of Rapid Amplification of cDNA Ends (RACE). The total length of the RhoprOctβ2-R (MF377526) cDNA sequence amplified was 1,799 bp yielding 447 amino acids with a molecular weight of 50,431 kDa (Figure 2A). Regions within the arrows indicate the predicted cDNA regions available on VectorBase (Figure 2A). The ORF of RhoprOctβ2-R spanned four exons with lengths of 455, 286, 353, and 599 bp separated by three intronic regions (Figure 2B). The length of the RhoprTyr1-R (MF377527) cDNA sequence amplified was 1,496 bp resulting in 455 amino acids with a molecular weight of 51,925 kDa (Figure 3A). The cDNA region within the first arrow and the second arrow + the third and the fourth arrow indicate predicted sequences obtained from VectorBase (Figure 3A). The RhoprTyr1-R ORF spanned a single exon with a length of 1,526 bp (Figure 3B). Furthermore, both receptors belong to the rhodopsin-like (Class A) GPCR superfamily characterized by seven transmembrane hydrophobic domains (TM), a DRY residue in the TM3 and an NPxxY motif in TM7 (Rovati et al., 2007; Rosenbaum et al., 2009). The amino terminal of RhoprOctβ2-R consisted of 65 amino acids compared to 49 amino acids for the RhoprTyr1-R (Figures 2, 3). The carboxyl terminal of RhoprOctβ2-R consisted of 66 amino acids vs. only 18 amino acids for RhoprTyr1-R. Characteristic to all Tyr1-Rs, the third intracellular loop of RhoprTyr1-R (151 amino acids) is elongated when compared to RhoprOctβ2-R's (70 amino acids) third intracellular loop (Figures 2A, 3A). In conclusion, the cloned cDNA sequences encode amino acid sequences predicted to form two GPCRs, RhoprOctβ2-R and RhoprTyr1-R. These newly found GPCRs contain all the structural features predicted to be required for function.

### Phylogenetic and sequence analysis

**Figure 4:**
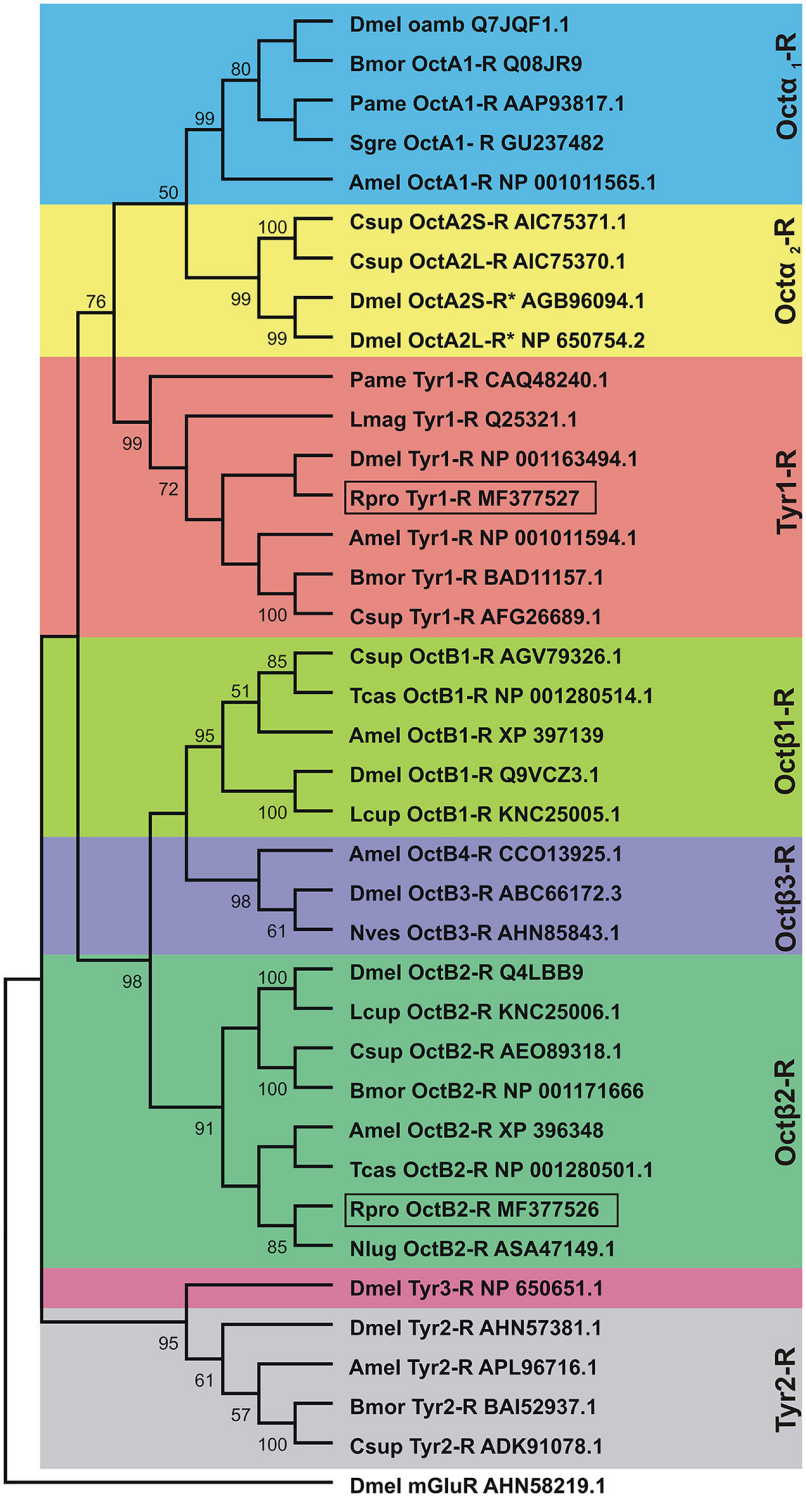
Phylogenetic tree of insect octopamine and tyramine receptors analyzed by Maximum Likelihood method using the JTT matrix-based model. Short form and GenBank accession numbers are indicated for each species. The percent bootstrap (1,000 replicates) support for the associated taxa that clustered together is shown next to the branches. Note that the cloned *R. prolixus* (Rpro) Octβ2-R and Tyr1-R are boxed. Taxonomic units (Dmel OctA2S-R and Dmel OctA2L-R) marked with asterisks indicate predicted sequences. The outgroup is *D. melanogaster* metabotropic glutamate receptor (Dmel mGlutR). Dmel, *Drosophila melanogaster*; Bmor, *Bombyx mori*; Pame, *Periplaneta americana*; Sgre, *Schistocerca gregaria*; Amel, *Apis mellifera*; Csup, *Chilo suppressalis*; Lmag, *Locusta migratoria*; Lcup, *Lucilia cuprina*; Tcas, *Tribolium castaneum*; Nves, *Nicrophorus vespilloides*; Nlug, *Nilaparvata lugens*.

**Figure 5:**
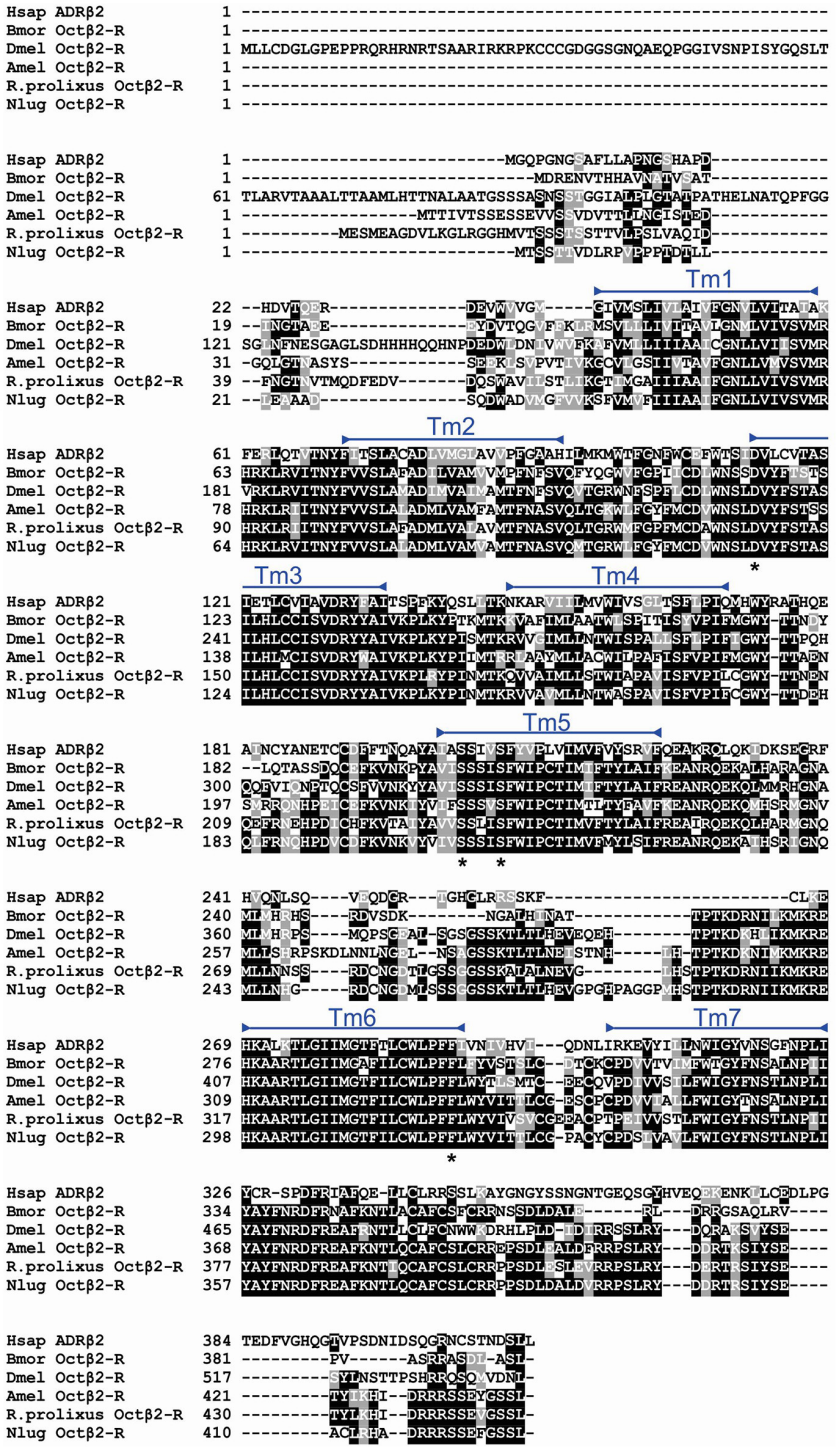
Multiple sequence alignment of insect Octβ2-Rs generated by MUSCLE alignment tool. Identical and similar amino acids across 60% of the sequences are shaded in black and gray, respectively. RhoprOctβ2-R transmembrane domains are highlighted with blue bars. Amino acids noted with asterisks below the alignment are signature residues conserved in adrenergic and adrenergic-like receptors. Dmel, *Drosophila melanogaster* (Q4LBB9); Bmor, *Bombyx mori* (NP_001280501.1); Csup, *Chilo suppressalis* (AEO89318.1); Nlug, *Nilaparvata lugens* (ASA47149.1); Hsap, *Homo sapiens*, ADRβ2, adrenergic receptor beta-2 (P07550.3).

**Figure 6:**
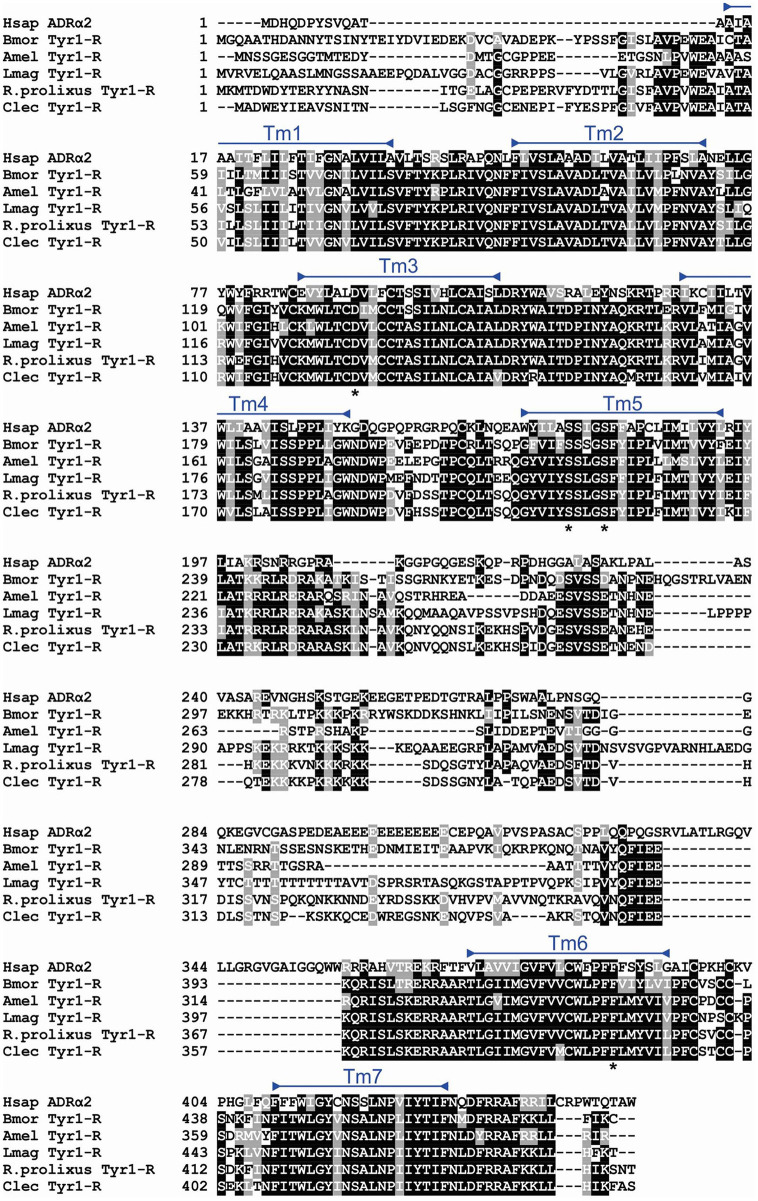
Multiple sequence alignment of insect Tyr1-Rs generated by MUSCLE alignment tool. Identical and similar amino acids across 60% of the sequences are shaded in black and gray, respectively. The RhoprTyr1-R transmembrane domains are highlighted with blue bars. Amino acids noted with asterisks below the alignment are signature residues conserved in adrenergic and adrenergic-like receptors. Bmor, *Bombyx mori* (BAD11157.1); Amel, *Apis mellifera* (NP_001011594.1); Lmag, *Locusta migratoria* (Q25321.1); Clec, *Cimex lectularius* (XP_014240675.1); Hsap, *Homo sapiens*, ADRα2, adrenergic receptor alpha-2 (AAA51666.1).

In order to determine the correct designation of the two receptors cloned, phylogenetic analysis was conducted with various insect octopamine and tyramine receptors. Different receptor types form separate monophyletic groups (Figure 4). Octβ-Rs form a large monophyletic group with each subtype of Octβ-Rs forming a separate monophyletic group. The Octα-Rs and the Tyr1-Rs form the other major monophyletic group. The single *D. melanogaster* Tyr3-R is part of Tyr2-R monophyletic group (Figure 4). RhoprOctβ2-R shared 78% identity and 87% similarity with *N. lugens's* Octβ2-R (ASA47149.1). RhoprTyr1-R shared 56% identity and 64% similarity with Tyr1-R of *D. melanogaster* (NP_001163494.1). Now that the correct designations have been established for both receptors, it is essential to consider the predicted biochemical features of these GPCRs. Post-translational modifications such as phosphorylation, N-glycosylation and cysteine palmitoylation of the receptors are essential for the structural integrity and functionality of the receptors (Kristiansen, 2004). RhoprOctβ2-R is predicted to be N-glycosylated at two sites at Asn^40^ and Asn^43^ (Figure 2A). Multiple serine and threonine residues are predicted to be phosphorylated by kinases in the third intracellular loop and the C-terminus (Figure 2A). For RhoprTyr1-R, N-glycosylation is noted at two sites, Asn^14^ and Asn^17^, at the N-terminus (Figure 3A). Multiple phosphorylation sites are highlighted in the long third intracellular loop between TM5 and TM6 (Figure 3A). Multiple sequence alignment of octopamine and tyramine receptors yielded key amino acids common in all insect octopamine and tyramine receptors (Figures 5, 6). In short, RhoprOctβ2-R and RhoprTyr1-R contain the predicted biochemical sites suggesting that these receptors are functionally viable and are closely related to other insect Octβ2 and Tyr1 receptors.

### Functional characterization of the receptors

**Figure 7:**
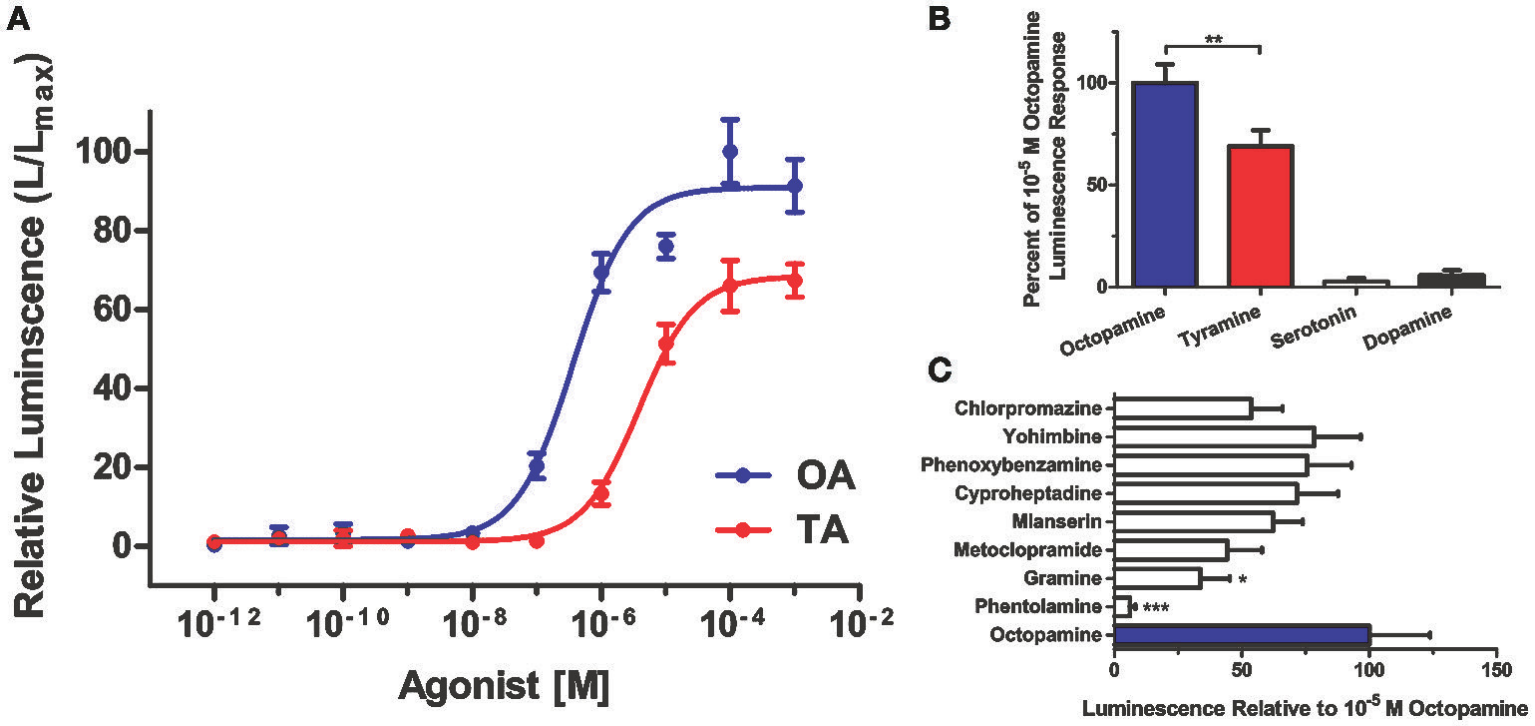
Functional characterization of *R. prolixus* Octβ2 receptor in HEK293 cells. **(A)** Dose-response curve showing the effects of octopamine (OA) and tyramine (TA) on RhoprOctβ2-R. **(B)** RhoprOctβ2-R is selective for octopamine and tyramine, while serotonin and dopamine are inactive against the receptor. **(C)** The effects of various biogenic amine antagonists on RhoprOctβ2-R. Phentolamine and gramine significantly reduced octopamine-induced receptor activation (One-Way ANOVA followed by Dunnett's Multiple Comparison Test compared to octopamine groups at 100%, ^*^P < 0.05, ^**^P < 0.01, ^***^P < 0.001). All receptor agonists and antagonists were tested at 10^−5^ M. Data represent the mean ± SEM of *n* = 5–6 per treatment.

**Figure 8:**
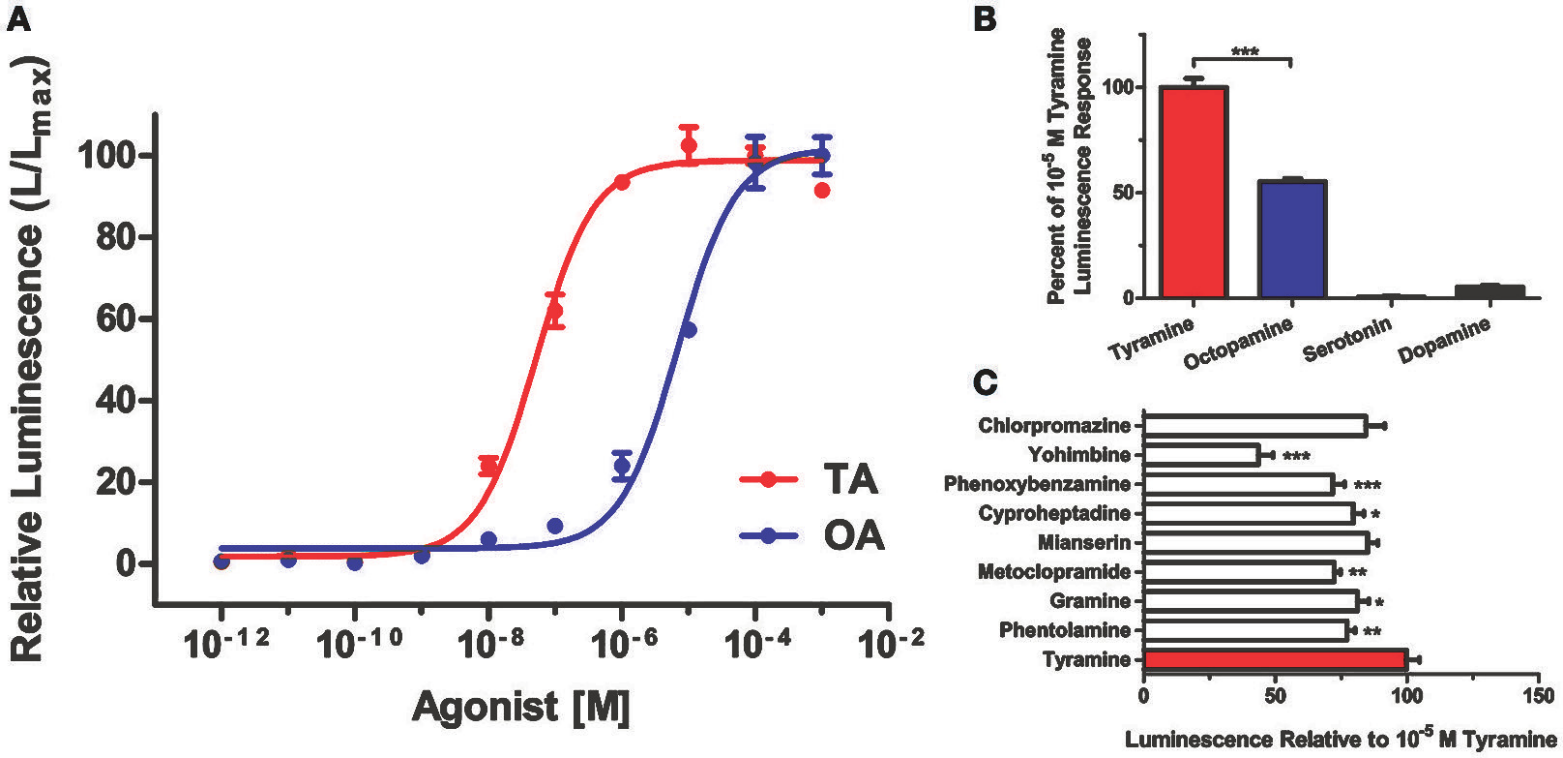
Functional characterization of *R. prolixus* Tyr1 receptor in HEK293 cells. **(A)** Dose-response curve showing the effects of tyramine (TA) and octopamine (OA) on the RhoprTyr1-R. **(B)** RhoprTyr1-R is more selective for tyramine. **(C)** Various biogenic amine antagonists, notably yohimbine and phenoxybenzamine, reduced the tyramine-induced activation of the RhoprTyr1-R (One-Way ANOVA followed by Dunnett's Multiple Comparison Test compared to tyramine groups at 100%, ^*^P < 0.05, ^**^P < 0.01, ^***^P < 0.001). All receptor agonists and antagonists were tested at 10^−5^ M. Data represents the mean ± SEM of *n* = 3–6 per treatment.

It was important to confirm the identity of RhoprOctβ2-R and RhoprTyr1-R by testing the activation of the receptors to their corresponding ligands. This was done by transfecting and transiently expressing both receptors in HEK293/CNG cells. The interaction of the ligand with the receptor was monitored by measuring the bioluminescence released due to calcium mobilization in the cytosol. Maximum activation of the receptors, peak luminescence, was detected within 5–10 s. RhoprOctβ2-R was activated in a dose-dependent manner by both octopamine and tyramine (Figure 7A). Tyramine (EC_50_ = 3.85 × 10^−6^ M) was ten times less potent than octopamine (EC_50_ = 3.67 × 10^−7^ M) in activating RhoprOctβ2-R. Tyramine was a partial ligand of RhoprOctβ2-R with a 69.00 ± 7.94% luminescence response relative to 10^−5^ M octopamine (One-Way ANOVA followed by Dunnett's Multiple Comparison Test compared to the octopamine group at 100%, ^**^P < 0.01) (Figure 7B). Other biogenic amines (serotonin and dopamine) were inactive against RhoprOctβ2-R, confirming its selectivity for octopamine and tyramine (Figure 7B). For the antagonists, phentolamine and gramine significantly reduced the 10^−5^ M octopamine-induced luminescence response, whereas partial inhibition was noted with chlorpromazine, mianserin and metoclopramide (One-Way ANOVA followed by Dunnett's Multiple Comparison Test compared to the octopamine group at 100%, ^*^P < 0.05, ^***^P < 0.001) (Figure 7C). Furthermore, tyramine and octopamine each activated RhoprTyr1-R in a dose-dependent manner with a threshold in the nanomolar range for tyramine (Figure 8A). Tyramine (EC_50_ = 5.17 × 10^−8^ M) was ~100 times more potent that octopamine (EC_50_ = 6.88 × 10^−6^ M). Analysis of various biogenic amine agonists (serotonin and dopamine) against RhoprTyr1-R revealed that RhoprTyr1-R is selective for tyramine and octopamine (yields ~50% of 10^−5^ M tyramine luminescence response) (Figure 8B). Various biogenic amine antagonists were effective in significantly reducing tyramine's luminescence response, most notably, yohimbine (>50% reduction in luminescence) and phenoxybenzamine (One-Way ANOVA followed by Dunnett's Multiple Comparison Test compared to the tyramine group at 100%, ^*^P < 0.05, ^**^P < 0.01, ^***^P < 0.001) (Figure 8C). HEK293/CNG cells transfected with empty vectors resulted in a luminescence response that was identical to control wells. As predicted, RhoprOctβ2-R and RhoprTyr1-R are functional GPCRs. Octopamine and tyramine bind to RhoprOctβ2-R and RhoprTyr1-R causing modification in second messengers.

### RhoprOctβ2-R and RhoprTyr1-R transcript expression

**Figure 9:**
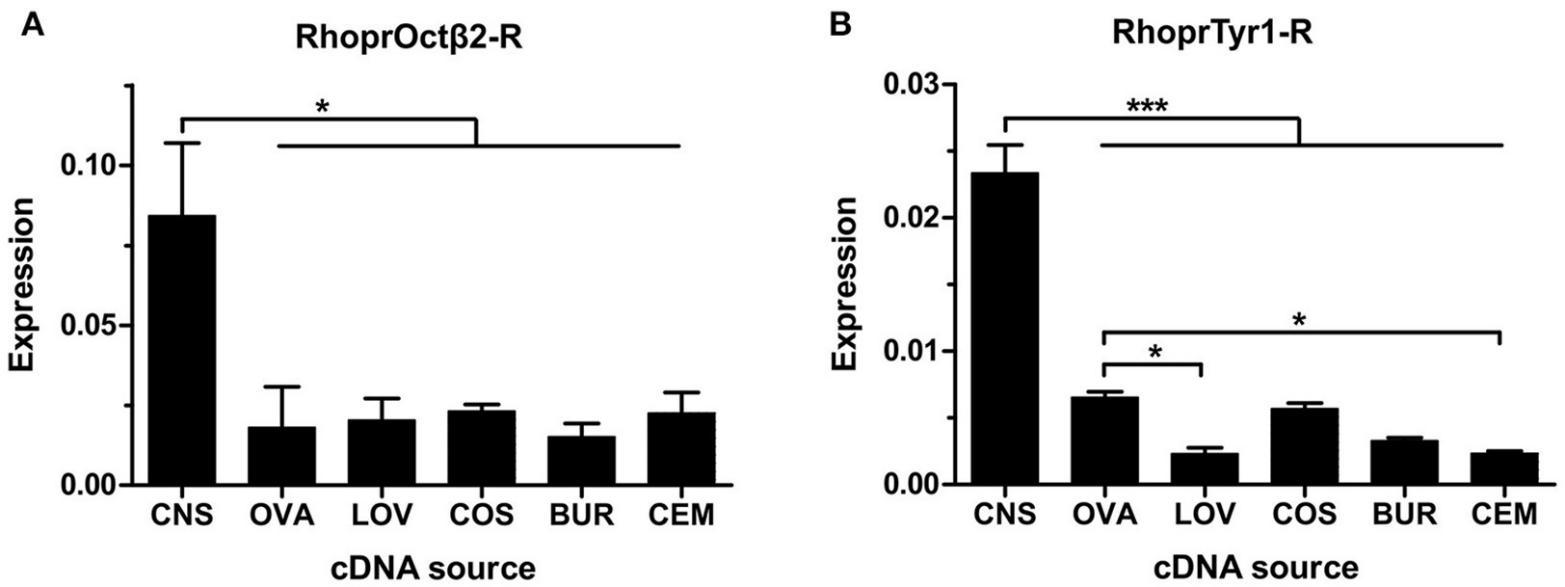
Spatial analysis of transcript expression of biogenic amine receptors in the adult female reproductive system. **(A)** RhoprOctβ2-R transcript is widely distributed in the reproductive tissues. The expression of RhoprOctβ2-R is significantly higher than the reproductive tissues (one-way ANOVA followed by Tukey multiple comparisons test; ^*^P < 0.05). **(B)** RhoprTyr1-R transcript is expressed in all reproductive tissues. RhoprTyr1-R transcript expression in the ovary is significantly higher than the lateral oviducts and the cement gland (one-way ANOVA followed by Tukey multiple comparisons test; ^*^P < 0.05, ^***^P < 0.001). CNS, central nervous system; OVA, ovary; LOV, lateral oviduct; COS, common oviduct and spermatheca; BUR, bursa; CEM, cement gland. Data represents the mean ± SEM of three biological replicates for RhoprOctβ2-R and four biological replicates for RhoprTyr1-R, each biological replicate included two technical replicates.

The expression of both receptors was analyzed in the CNS and in the reproductive system of adult female *R. prolixus* using Real Time Quantitative PCR. RhoprOctβ2-R transcript expression was highly expressed in the CNS relative to the female adult reproductive tissues (Figure 9A). RhoprOctβ2-R expression was roughly similar in all reproductive tissues (Figure 9A). Slightly low expression of RhoprOctβ2-R transcript was found in the bursa compared to other reproductive tissues (Figure 9A). Transcript distribution of RhoprTyr1-R was enriched in the CNS relative to adult female reproductive tissues (Figure 8B). The expression of RhoprTyr1-R in the ovary and common oviduct + spermetheca was similar (Figure 9B). Low RhoprTyr1-R expression was noted in the lateral oviducts and the cement gland (Figure 9B). Overall, the expression of RhoprOctβ2-R and RhoprTyr1-R in the reproductive system suggests that octopamine and tyramine could utilizing these receptors to cause a modification in the rhythmic contractions of the reproductive visceral muscle (Hana and Lange, 2017). In fact, tyramine's lack of direct action at the oviducts could be substantiated by the lower expression of RhoprTyr1-R transcript relative to other tissues (Hana and Lange, 2017).

## Discussion

Two receptors, Octβ2-R and Tyr1-R, have been cloned and characterized in *R. prolixus*. These receptors were deorphaned and their pharmacological profiles were analyzed. Previously, we have shown that octopamine, acting via cAMP, decreases the amplitude of spontaneous oviduct contractions, whereas tyramine was ineffective (Hana and Lange, 2017). This previous data complements the present data that reveals RhoprOctβ2-R couples to a G_s_ protein leading to the activation of adenylate cyclase and elevation of intracellular cAMP in HEK293/CNG cells in the functional receptor assay. The increase in cAMP in HEK293/CNG cells would have in-turn opened the CNG channel resulting in an influx of Ca^2+^ from the extracellular medium. Intracellular Ca^2+^ levels were detected by the reporter molecule aequorin from the hydrozoan *Aequorea victoria*. RhoprTyr1-R likely couples to a G_q_ protein leading to the release of Ca^2+^ from intracellular stores through the IP_3_ pathway which would then also have been detected with the reporter aequorin.

RhoprOctβ2-R and RhoprTyr1-R share key structural features similar to other insect Octβ2-Rs and Tyr1-Rs. The third intracellular loop of both receptors were elongated relative to other loops, in terms of comparison, Tyr1-Rs are known to have a lengthy third intracellular loop as seen with RhoprTyr1-R. Interestingly, multiple phosphorylation sites (S and T) are found in the third intracellular loops for all octopamine and especially tyramine receptors. Phosphorylation of these residues likely leads to receptor signaling and desensitization (Kristiansen, 2004). As shown from the sequence alignment, there are key amino acids in all receptors that are important for ligand binding and are widely conserved in vertebrate and invertebrate receptors. For example, for RhoprOctβ2-R, the Asp^142^ residue in TM3, Ser^231^ and Ser^235^ in TM5 and Phe^338^ in TM6 are all believed to participate in ligand binding (Strader et al., 1995; Sato et al., 1999; Blenau and Baumann, 2001; Huang et al., 2007; Chen et al., 2011). In 2011, Chen and colleagues reported that an Asp^115^, Ser^202^, and Tyr^300^ were required for *Bombyx mori* Octβ2-R activation and cAMP elevation in HEK293 cells (Chen et al., 2011). Similarly, a report by Ohta et al. (2004) showed that the Asp^134^ residue in TM3 and Ser^218^ and Ser^222^ in TM5 were essential for activation of the *B. mori* Tyr1-R suppression of cAMP levels in HEK293 cells (Ohta et al., 2004). Essentially, these ligand interacting residues found in RhoprOctβ2-R and RhoprTyr1-R are homologous to the residues found in other octopamine and tyramine receptors from other insects. Therefore, it is established that the cloned receptors contain the predicted structural and biochemical features needed for biological activity.

Predictions are hypothetical and do not grant function, therefore, biological activity of Octβ2-R and RhoprTyr1-R was investigated in HEK293/CNG cells. Octopamine is one order of magnitude more potent than tyramine in activating RhoprOctβ2-R. The half-maximal activation was 3.67 × 10^−7^ M for octopamine compared to 3.85 × 10^−6^ M for tyramine. Octopamine fully activated the receptor, unlike tyramine which is a partial agonist. Half-maximal activation values (EC_50_) of other cloned Octβ2-Rs vary with a range of 10^−9^ to 10^−7^ M. Octopamine has been shown to be one order of magnitude more potent than tyramine in *N. lugens* (Wu et al., 2017), *Apis mellifera* (Balfanz et al., 2014) and *D. melanogaster* (Maqueira et al., 2005). In *Chilo suppressalis*, octopamine was found to be two to three orders of magnitude more potent than tyramine (Wu et al., 2012). RhoprOctβ2-R was antagonized by phentolamine > gramine > metoclopramide. In general mianserin and phentolamine have been shown to be effective antagonists of Octβ2-Rs in other insects (Maqueira et al., 2005; Wu et al., 2012, 2017; Balfanz et al., 2014); however, this is not always the case, and chlorpromazine and metoclopramide antagonized the *B. mori* Octβ2-R (Chen et al., 2010). Octopamine's potency in activating Octβ2-R varies with different insects. Similarly, functional analysis of RhoprTyr1-R showed that tyramine was significantly more potent than octopamine in activating the receptor. Tyramine (EC_50_ = 5.17 × 10^−8^ M) was found to be two orders of magnitude more potent than octopamine (EC_50_ = 6.88 × 10^−6^ M). Nonetheless, octopamine fully activated Tyr1-R at ≥10^−4^ M concentrations. Tyr1-Rs from *B. mori* (Ohta et al., 2003) and *L. migratoria* (Vanden Broeck et al., 1995; Poels et al., 2001) have been shown to be activated by tyramine at similar concentrations reported here for RhoprTyr1-R. Tyramine was two orders of magnitude more potent than octopamine in these organisms (Vanden Broeck et al., 1995; Poels et al., 2001; Ohta et al., 2003). In *A. mellifera* (Blenau and Baumann, 2001) and *D. melanogaster* (Saudou et al., 1990; Enan, 2005), tyramine was only one order of magnitude more potent than octopamine. Interestingly, tyramine is three times more potent than octopamine in activating a Tyr1-R in *C. suppressalis* (Wu et al., 2013). Yohimbine was the most effective antagonist in inhibiting tyramine's activation of RhoprTyr1-R. Overall, yohimbine has been established as the most potent antagonist of insect Tyr1-Rs (Arakawa et al., 1990; Saudou et al., 1990; Robb et al., 1994; Vanden Broeck et al., 1995; Poels et al., 2001; Enan, 2005; Rotte et al., 2009). The contribution of different cell lines and the type of expression (stable and transient) can of course alter the pharmacological data obtained in these assays. Altogether, RhoprOctβ2-R and RhoprTyr1-R are bioactive and exhibit a distinct yet similar pharmacological profile compared to other octopamine and tyramine receptors.

RhoprOctβ2-R and RhoprTyr1-R are confirmed to be active receptors, but where and what physiological processes could these receptors mediate? Analysis of Octβ2-R and Tyr1-R transcript distribution reveals that both receptors are highly expressed in the CNS (Blenau et al., 2000; Rotte et al., 2009; Wu et al., 2012, 2013, 2017; El-Kholy et al., 2015). Octβ2-R transcript is highly expressed in skeletal muscle, reproductive organs, leg, antenna and other structures (El-Kholy et al., 2015; Wu et al., 2017) while Tyr1-R transcript is strongly expressed in the heart and minorly expressed in the reproductive organs (El-Kholy et al., 2015). Similar to other insects, strong expression of RhoprOctβ2-R and RhoprTyr1-R transcripts was detected in the CNS relative to the transcript expression in adult female reproductive system. RhoprOctβ2-R transcript expression is similar to Octβ2-R transcript expression in *D. melanogaster* and *N. lugens* in the reproductive system (Lim et al., 2014; Li et al., 2015; Wu et al., 2017). Indeed, the differential expression of RhoprOctβ2-R and RhoprTyr1-R transcripts in the reproductive system are likely correlated with the physiological effects observed (Hana and Lange, 2017). Previously, it was shown that an Octβ receptor is responsible for the inhibitory actions in the oviducts and the bursa (Hana and Lange, 2017). The expression of RhoprOctβ2-R transcript in the oviducts and the bursa supports this hypothesis, in fact, it is further strengthened due to phentolamine strongly antagonizing RhoprOctβ2-R. On the other hand, lower RhoprTyr1-R transcript expression, relative to other reproductive tissues, reinforces the fact that tyramine is unable to inhibit oviduct contractions by itself (Hana and Lange, 2017). RhoprTyr1-R transcript expression at the lateral oviducts could signify tyramine's neuromodulator rather than neurotransmitter properties at the oviducts (Hana and Lange, 2017). In this scenario, tyramine modulates the activity of other neuropeptides that stimulate contraction rather than directly influencing contraction. To summarize, octopamine and tyramine could be utilizing these found GPCRs to modify rhythmic contractions and other various female reproductive processes.

In conclusion, the cDNA of RhoprOctβ2-R and RhopTyr1-R has been cloned and functionally characterized. RhoprOctβ2-R isspecifically activated by octopamine, whereas RhoprTyr1-R is specifically activated by tyramine. The wide spatial distribution of these two receptor transcripts in the female reproductive system suggest their importance in modulating reproductive processes. Octβ2-R has already been established in *D. melanogaster* and *N. lugens* to be important for ovulation of eggs (Lim et al., 2014; Li et al., 2015; Wu et al., 2017). The RhoprOctβ2-R expressed in the oviducts of *R. prolixus* is likely involved in the relaxation of the oviducts which may be a vital step in the process of ovulation. RhoprOctβ2-R and RhoprTyr1-R knockdown studies are needed to further elucidate the role of these receptors in ovulation and other reproductive processes in *R. prolixus*.

## Ethics statement

Insects are not considered as animals for the purposes of animal care licenses by the Canadian Council of Animal Care and they do not require any animal protocols.

## Author contributions

AL: Principal investigator and edited the manuscript. AL, SH: Designed the experiments. SH: Conducted all experiments, analyzed and wrote the manuscript.

### Conflict of interest statement

The authors declare that the research was conducted in the absence of any commercial or financial relationships that could be construed as a potential conflict of interest.
