# Corrigendum: Cloning and Functional Characterization of Octβ2-Receptor and Tyr1-Receptor in the Chagas Disease Vector, *Rhodnius prolixus*

**DOI:** 10.3389/fphys.2020.00649

**Published:** 2020-06-30

**Authors:** Sam Hana, Angela B. Lange

**Affiliations:** Department of Biology, University of Toronto Mississauga, Mississauga, ON, Canada

**Keywords:** octopamine, tyramine, G-protein-coupled receptor, antagonists, insect

In the original article, there was an incomplete sentence in the Introduction where it was stated that “A and Octβ2-R knockdown hindered ovulation in *Nilaparvata lugens* (Wu et al., 2017).” The full sentence should read, “A recent study demonstrated Octβ2-R knockdown hindered ovulation in *Nilaparvata lugens* (Wu et al., 2017).” A correction has been made to the **Introduction**, paragraph 3:

“Octopamine and tyramine signaling pathways have been shown to be essential in modulating the reproductive system of various insects. For example, lack of tyramine and octopamine in *Drosophila melanogaster* (tyrosine decarboxylase 2 mutated flies) resulted in reproductive sterility due to egg retention (Cole et al., 2005). Insects that specifically lack octopamine (tyramine β-hydroxylase mutants) accumulated eggs in their ovaries due to abolished ovulation (Monastirioti et al., 1996; Monastirioti, 2003). A tyramine 1 (Tyr1) receptor in *Locusta migratoria*, the octopamine receptor in the mushroom bodies (OAMB) and octopamine beta 2 (Octβ2) receptor in *D. melanogaster*, have also been linked to reproductive physiology in both insects (Lee et al., 2003, 2009; Donini and Lange, 2004; Molaei et al., 2005; Lim et al., 2014; Li et al., 2015). A recent study demonstrated Octβ2-R knockdown hindered ovulation in *Nilaparvata lugens* (Wu et al., 2017). Octopamine and tyramine may exert some of their effects by influencing the contractions of the reproductive musculature. Thus, octopamine reduces the amplitude, frequency and basal tonus of lateral oviduct contractions in *D. melanogaster* (Middleton et al., 2006; Rodriguez-Valentin et al., 2006), *L. migratoria* (Lange and Orchard, 1986), and *Stomoxys calcitrans* (Cook and Wagner, 1992).”

In addition, there was a spelling in the Materials and Methods. The word “head-inactivated” was used instead of “heat-inactivated.” A correction has been made to the **Materials and Methods** section, subsection **Mammalian Expression Vectors and Transfection of the Receptors**, paragraph 2:

“A HEK293/CNG cell line that stably expresses a modified cyclic nucleotide-gated channel (CNG) (previously available from BD Biosciences, Mississauga, ON, Canada) were raised in Dulbecco's Modified Eagle Medium Nutrient Mixture F12-Ham (DMEM/F-12) (Thermo Fisher Scientific, Waltham, MA, USA) supplemented with 10% heat-inactivated fetal bovine serum, 1% penicillin and streptomycin, and 100 μg/mL G418. The cells were incubated at 37°C in 5% CO_2_. The cells were grown in T75 flasks to 90–95% confluency and were transiently co-transfected with either expression vector containing the receptor and aequorin at a 2:1 ratio (transfection reagent to expression vectors) using X-tremeGENE® HP DNA Transfection Reagent (Roche Applied Science, Penzberg, Germany). The cells were incubated for 72 h and used for the functional cell assay.”

The authors apologize for this error and state that this does not change the scientific conclusions of the article in any way. The original article has been updated.
