# Supplementary material for: Cloning and Functional Characterization of Octβ2-Receptor and Tyr1-Receptor in the Chagas Disease Vector, Rhodnius prolixus
Source: Front Physiol. 2017 Sep 26;8:744. doi: 10.3389/fphys.2017.00744 (PMC5623054; doi:10.3389/fphys.2017.00744)
Supplement: Supplementary file 1 [file Table1.DOCX]

***Supplementary Material***

**Cloning and Functional Characterization of Octβ2-Receptor and Tyr1-Receptor in the Chagas Disease Vector, *Rhodnius prolixus*.**

**Sam Hana*, Angela B. Lange**

*** Correspondence:** Sam Hana: sam.hana@mail.utoronto.ca

**Supplementary Tables**

**Table S1.** Primers for the amplification of the receptor fragments

| **Primer** | **Sequence (5'- 3')** |
| --- | --- |
| **RhoprOctβ2-R** |  |
| Octβ2-R Forward | GTACGACAGTGTTGCCAAGTTTG |
| Octβ2-R Reverse | ACTGCTGCCCACTTCGC |
| **RhoprTyr1-R** |  |
| Tyr1-R Forward | CTAATGAAAATGACTGACTGGGAC |
| Tyr1-R Reverse | GTATAAATAATTGGATTTAACGCCG |

**Table S2.** Primers for the amplification of 3’ region of the receptors

| **Primer** | **Sequence (5'- 3')** |
| --- | --- |
| **Plasmid-Specific Primers** |  |
| pDNR-LIB -25 REV | GTGGATAACCGTATTACCGCC |
| **RhoprOctβ2-R** |  |
| Octβ2-R 3'RACE For2 | TTCAAGAATACCATCCAGTGTGC |
| Octβ2-R 3'RACE For1 | ACCCGCAGCATATATTCAGAAAC |
| **RhoprTyr1-R** |  |
| Tyr1-R 3'RACE For4 | GATGTACATGTTCCTGTGATGGC |
| Tyr1-R 3'RACE For3 | GGTCCAAGTAAACCAGTTTATCGAAG |
| Tyr1-R 3'RACE For2 | GGAGAGACGAGCCGCAC |
| Tyr1-R 3'RACE For1 | CATGTACGTCATCTTACCCTTC |

**Table S3.** Primers for the amplification of 5’ region of the receptors

| **Primer** | **Sequence (5'- 3')** |
| --- | --- |
| **Plasmid-Specific Primers** |  |
| pDNR-LIB FOR1 | GTGGATAACCGTATTACCGCC |
| pDNR-LIB FOR2 | ACGGTACCGGACATATGCC |
| **RhoprOctβ2-R** |  |
| Octβ2-R 5'RACE Rev7 | CTAATGAAAATGACTGACTGGGAC |
| Octβ2-R 5'RACE Rev6 | TCTACATCTTCAAAATCTTGCATCG |
| Octβ2-R 5'RACE Rev5 | GGCAATAATGATCGCTCC |
| Octβ2-R 5'RACE Rev4 | CACGATCACCAACAGATTGC |
| Octβ2-R 5'RACE Rev3 | GATTATCCTCAACTTCCTGTGCC |
| Octβ2-R 5'RACE Rev2 | GAGCGATACGACGAAATAATTAGTG |
| Octβ2-R 5'RACE Rev1 | GAACGTCATCACAGCCAGC |
| **RhoprTyr1-R** |  |
| Tyr1-R 5'RACE Rev5 | GTCCCAGTCAGTCATTTTCATTAG |
| Tyr1-R 5'RACE Rev4 | GTTCGCCAGTTATGTTGCTTG |
| Tyr1-R 5'RACE Rev3 | CCATACTGGCACAGCGAATG |
| Tyr1-R 5'RACE Rev2 | GCTACAGTTAAATCAGCTACAGCTAACG |
| Tyr1-R 5'RACE Rev1 | CACACATGTATCCCAAATTCCCATC |

**Table S4.** Primers used for the mammalian expression vector preparation

| **Primer** | **Sequence (5'- 3')** |
| --- | --- |
| **Translation Initiation** |  |
| **RhoprOctβ2-R** |  |
| Octβ2-R (Kozak) For | **GCCACC**ATGGAAAGCATGGAGGCAG |
| Octβ2-R (Stop) Rev | CAGATCAAAGACTGCTGCCC |
| **RhoprTyr1-R** |  |
| Tyr1-R (Kozak) For | **GCCACC**ATGAAAATGACTGACTGGG |
| Tyr1-R (Stop) Rev | GGTCCAAGTAAACCAGTTTATCGAAG |
| **Insertion of Restriction Sites** |  |
| **RhoprOctβ2-R** |  |
| Octβ2-R (Bgl II) For | GATCTAGATCTGCCACCATGGAAAGCAT |
| Octβ2-R (Bam II) Rev | ATCATGGATCCTATATCAAAGACTGCTGCCCAC |
| **RhoprTyr1-R** |  |
| Tyr1-R (Bgl II) For | GATCTAGATCTGCCACCATGAAAATGACTG |
| Tyr1-R (Bam II) Rev | ATCATGGATCCCTTAAACATCACGTATTAGA  CTTAATATG |

**Table S5**. Primers used for RT-qPCR analysis of receptor transcripts.

| **Primer** | **Sequence (5'- 3')** |
| --- | --- |
| **RhoprOctβ2-R** |  |
| Octβ2-R qPCR Forward | GTACGACAGTGTTGCCAAGTTTG |
| Octβ2-R qPCR Reverse | CACGATCACCAACAGATTGC |
| **RhoprTyr1-R** |  |
| Tyr1-R qPCR Forward | GGAGAGACGAGCCGCAC |
| Tyr1-R qPCR Reverse | CTGATGGACAGCAAACCGAAC |
| **Rhopr-α-tubulin** |  |
| α-TUB Forward | GTGTTTGTTGATTTGGAACCTACAG |
| α-TUB Reverse | CCGTAATCAACAGACAATCTTTCC |
| **Rhopr-β-actin** |  |
| β-ACT Forward | AGAGAAAAGATGACGCAGATAATGT |
| β-ACT Reverse | ATATCCCTAACAATTTCACGTTCG |
| **Rhopr-ribosomal protein 49** |  |
| Rp49 Forward | GTGAAACTCAGGAGAAATTGGC |
| Rp49 Reverse | AGGACACACCATGCGCTATC |
